# The Role of Environmental Reservoirs in Human Campylobacteriosis

**DOI:** 10.3390/ijerph10115886

**Published:** 2013-11-08

**Authors:** Harriet Whiley, Ben van den Akker, Steven Giglio, Richard Bentham

**Affiliations:** 1 Environmental Health, School of the Environment, Flinders University, P.O. Box 2100, Adelaide 5001, South Australia, Australia; E-Mail: richard.bentham@flinders.edu.au; 2 School of Chemical Engineering, The University of Adelaide, Adelaide 5001, South Australia, Australia; E-Mail: Ben.vandenAkker@adelaide.edu.au; 3 Healthscope Pathology, South Australia, 1 Goodwood Rd., Wayville 5034, South Australia, Australia; E-Mail: Steven.Giglio@healthscope.com.au

**Keywords:** campylobacteriosis, *Campylobacter* spp., *C. jejuni*, environmental reservoirs, risk assessment

## Abstract

Campylobacteriosis is infection caused by the bacteria *Campylobacter* spp. and is considered a major public health concern. *Campylobacter* spp. have been identified as one of the most common causative agents of bacterial gastroenteritis. They are typically considered a foodborne pathogen and have been shown to colonise the intestinal mucosa of all food-producing animals. Much emphasis has been placed on controlling the foodborne pathway of exposure, particularly within the poultry industry, however, other environmental sources have been identified as important contributors to human infection. This paper aims to review the current literature on the sources of human exposure to *Campylobacter* spp. and will cover contaminated poultry, red meat, unpasteurised milk, unwashed fruit and vegetables, compost, wild bird faeces, sewage, surface water, ground water and drinking water. A comparison of current *Campylobacter* spp. identification methods from environmental samples is also presented. The review of literature suggests that there are multiple and diverse sources for *Campylobacter* infection. Many environmental sources result in direct human exposure but also in contamination of the food processing industry. This review provides useful information for risk assessment.

## 1. Introduction

*Campylobacter* spp. are the most common cause of acute bacterial enteritis in humans [1,2,3]. They are typically considered a foodborne pathogen and have been identified as the leading cause of food poisoning in Europe [4], the United States [5], Canada [6] and Australia [7].

Campylobacteriosis refers to disease as a result of *Campylobacter* spp. infection [3]. The most common cause of human infection is *Campylobacter jejuni*, followed by *Campylobacter coli*, but *Campylobacter lari*, *Campylobacter fetus* and *Campylobacter upsaliensis* have also been reported to cause human infections [3,4,8]. Commonly reported symptoms of campylobacteriosis include diarrhoea, abdominal pain, fever, malaise and headaches [9]. Although it is generally self-limiting, approximately one tenth of laboratory confirmed cases require hospitalisation [10]. There are also some rare complications associated with *Campylobacter* infection. Guillain-Barré syndrome (GBS) is estimated to occur in one out of 1,000 cases of *C. jejuni* infection. It is a disease of the nervous system which can result in acute neuromuscular paralysis [11]. Reiter’s syndrome affects approximately 1% of campylobacteriosis causes. It is a reactive arthritis that can affect multiple joints causing pain and incapacitation [9]. Irritable bowel syndrome is anther sequel to campylobacteroisis that causes significant social and economic burden [11].

*Campylobacter* spp. are capable of zoonotic transfer through the faecal-oral route and have been reported to be unable to multiply outside warm-blooded host animals [9,12,13]. They colonise the intestinal mucosa of all food-producing animals and humans. However the favoured environmental niche is considered to be the intestinal tract of all avian species [14]. Thus, the primary risk factor for *Campylobacter* infection is considered to be exposure to be contaminated food of poultry origin [15,16,17].

Although *Campylobacter* spp. are unable to multiply outside a host, they can survive in different environmental sources [1]. The survival time is depended on the species and the environmental conditions including temperature, light, biotic interactions, oxygen and nutrient concentrations [18]. These environmental sources are also considered important contributors to human infection and include soil, manure, aquatic environments and water sources. The precise role that each environmental source plays in the complex epidemiology of *Campylobacter* infection is still unknown [19,20,21]. This review will therefore explore the current knowledge about environmental sources of *Campylobacter* spp.

## 2. Foodborne Pathogen

### 2.1. Poultry

Campylobacteriosis is largely considered as a foodborne disease with poultry considered the principle vehicle of transmission. Studies have identified eating or handling raw or undercooked chicken as a major risk factor campylobacteriosis in humans [11,13,16,17]. The percentage of human campylobacteriosis cases that are attributed to eating or handling raw poultry varies between countries and studies. Estimates of cases that have a foodborne origin range from 30% [22] to 58%–76% [17] and up to 80% may be attributed to the chicken reservoir as a whole [11]. In 2008, Stafford *et al.* estimated that 75% of campylobacteriosis was foodborne [15]. In 2009 Gillespie published a rebuttal of Stafford *et al.* stating they over-estimated the role of chicken consumption in cases of campylobacteriosis by a factor of 3.4 [23]. Often the source of notified cases is unable to be determined, which means that the actual number of foodborne cases is unknown [24].

### 2.2. Poultry Production

The percentage of chickens contaminated with *Campylobacter* spp. varies between countries. In the United States studies indicate that nearly 90% of flocks are colonised [25] which is supported by data from a European Union study that found and average 71.2% of broiler batches and 75.8% of broiler carcasses to be contaminated with *Campylobacter spp.* [26]. However, national surveys in Sweden indicate that less than 10% of broilers are contaminated with *Campylobacter* spp. It remains to be seen if there is similar variability throughout other regions of the World [14]*.*

There are a range of environmental sources poultry are exposed to both on the farm and at the processing plant that can result in contamination with *Campylobacter* spp*.* The spread of *Campylobacter* spp. throughout flocks is extremely rapid, particularly amongst hatchlings. Studies have shown that three days contact with a single *Campylobacter* spp. infected bird is sufficient for the majority of the flock to be colonised [27]. Pearson *et al.* [28] also suggests that vertical transmission of *Campylobacter* spp. from breeder flocks to offspring is a source of contamination, but this is not widely accepted [5]. Horizontal transmission can occur via contaminated water, litter, faecal contact and other vectors such as insects [29,30], rodents and farm personnel [25]. Feed has not been implicated in the spread of *Campylobacter* spp. as it is too dry to facilitate its survival [31].

### 2.3. Poultry Processing

Within processing plants cross contamination is a significant problem particularly when *Campylobacter* spp. free flocks follow contaminated flocks [13]. Contaminated poultry that enters the processing plant can contain *Campylobacter* spp. populations ranging from 10^5^ to 10^8^ CFU/g of faeces. These high levels allow the bacteria to be easily spread throughout the plant [31]. The scalding and defeathering procedures have the potential for cross-contamination. *Campylobacter* spp. has periodically been removed from scald water and it has been postulated that the opening and closing of follicles may allow the retention of *Campylobacter* spp. within the carcass [25]. The use of recycled water throughout processing plant is another procedure that results in cross contamination. *Campylobacter* spp. may also be transported throughout a processing plant by personnel moving from one area of the plant to another [31]. 

Due to the numerous opportunities for cross contamination, processing plants employ a variety of physical, chemical and irradiation based treatments to reduce the microbial contamination of poultry carcasses [31,32]. These treatments significantly reduce the levels of *Campylobacter* spp. but they are unable to achieve complete removal and since dose levels as low as 500 organisms have been reported to cause illness, the contaminated carcasses will still pose a threat to public health [31]. Contaminated poultry can also result in cross contamination to other reservoirs, including food produce [33] and natural waters [34].

### 2.4. Other Foodborne Pathways

Consumption of unpasteurised milk and raw red meat, fruits and vegetables have also been identified as sources of foodborne campylobacteriosis in humans [35,36,37]. *Campylobacter* spp. may be present in milk from faecal contamination during the milking process or an udder infection [37]. Unpasteurised milk was first identified as a source of human campylobacteriosis in 1978 when four cases of *C. fetus* infection were identified within a three week period in a hospital in Los Angeles County. Three of the four patients had drunk large quantities of an identical brand of commercially available certified raw milk and had *C. fetus* subspecies *jejuni* isolated from their blood. A telephone survey, that was conducted to compare cases to controls, identified that the consumption of raw milk was a confirmed risk factor of *C. fetus* infection [38]. Information from two outbreaks of campylobacteriosis associated with drinking unpasteurised milk, one from the United Kingdom [39] and one from the Netherlands [40] have been used to derived a *C. jejuni* dose response model (Equation (1)) [41]:

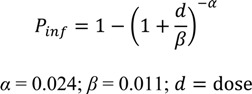
(1)


A recent study from the United States found that 5% (12/262) of campylobacteriosis outbreaks from 1997–2008 were due to consumption of contaminated pork, beef or game [42]. In Europe, molecular typing studies of *C. jejuni* isolates from cattle have demonstrated a similarity with human strains [43,44]. Sporadic outbreaks of campylobacteriosis have been linked to contaminated red meat. In 1980 an outbreak in Dutch military barracks was associated with contaminated steak tartare and in 1979 an outbreak in a Japanese daycare centre was confirmed by culture to be caused by contaminated pork [45]. In 2004 a study in Canada investigated the prevalence of *Campylobacter* spp. throughout a cattle ranch. Sixty cows were tested over a 4-month period for the presence of *Campylobacter* spp. shed in faeces. The cows were restricted to individual pens to minimise transmission between animals. During the study, every cow tested positive for *Campylobacter* spp. at least once, although the survival time of *Campylobacter* spp. once excreted was not investigated [35].

Verhoeff-Bakkenes *et al.* identified consumption of raw fruits and vegetables contaminated with faecal matter as a possible source of campylobacteriosis [36]. In a study conducted in The Netherlands they found 13 out of 5,640 fruit and vegetables samples to be *Campylobacter* spp. positive, giving a prevalence of 0.23%. Packaged fruit and vegetables had a significantly higher prevalence of *Campylobacter* spp. (0.36%) when compared to unpackaged products (0.07%). However, recent case-control studies designed to identify risk factors for campylobacteriosis have found the consumption of fruit as a protective factor and not a risk [11,46].

### 2.5. Public Perception and Food Safety

Consumer knowledge on the potential risks associated with food preparation has been identified as a determining factor of foodborne infection [47]. In England and Wales it is estimated that at least 60 per cent of food poisoning is acquired in the home. Consumers are not fully aware of all the risks associated with food preparation and believe that the responsibility lies instead with food manufacturers [48]. Information provided to consumers on food safety including information on the dangers involved with raw meats, particularly the risk of cross-contamination and correct food preparation practices could help decrease the risk of foodborne campylobacteriosis [49]. 

## 3. Animal Vectors

### 3.1. Domestic Animals

A range of domestic animals have been identified as hosts for *Campylobacter* spp. [20,50,51,52]. In 1999 Baker *et al.* identified domesticated dogs and cats as a potential source of human campylobacteriosis with 55% (108/195) of cats and 49% (143/289) of dogs testing positive to *Campylobacter* spp. [53]. Further studies conducted on domestic dogs in Canada found that 58% (39/70) of healthy dogs’ faeces and 97% (63/65) of the diarrheic dogs’ faeces contained detectable levels of *Campylobacter* spp. Positive samples contained between 10^3^ and 10^8^ *Campylobacter* spp. per gram of faeces. In domestic dogs and cats, intensive housing and open drains have been shown to increase the risk of *Campylobacter* spp. carriage by 2 and 2.6 times, respectively. Feeding of raw meat to dogs has also been identified as a risk factor for dogs to become carriers of *Campylobacter* spp. [51]. Another study found *C. jejuni* in 5/70 (7%) healthy domestic dog faeces and 30/65 (46%) in diarrheic domestic dog faeces at concentrations up to 10^6^ organisms/g [52]. As the infectious dose of *C. jejuni* is estimated to be 500 organisms [54], these high concentrations present in the faeces pose a risk for accidental exposure and possibly infection. A recent study by Gras *et al.* found 132/687 (19%) of domestic dogs and cat stools to be positive for *Campylobacter* spp. The detected *C. jejuni* and *C. coli* multilocus sequence type (STs) from pets and owners were compared. There were 2/68 (2.94%) cases where owner was infected with an identical ST to their pet (compared 0.134/68 (0.2%) expected to occur by chance). This study identified dog ownership, particularly puppy ownership, as a significant increase in risk for campylobacteriosis [55]. 

### 3.2. Wild Animal Faeces

The shedding of wild birds faeces into the environment has been identified as a significant reservoir of *Campylobacter* spp. [56]. Exposure to contaminated wild bird faeces in playgrounds has been recognised as an emerging environmental source of campylobacteriosis, particularly for children [19]. The frequent hand to mouth behaviour associated with children provides a mechanism for ingesting campylobacters [57]. Many playgrounds are natural habitats for a range of wild animals including birds, lizards, and stray cats and dogs. A New Zealand study tested avian faecal matter found in children’s playgrounds and discovered that a total of 12.5% (24/192) were positive for *C. jejuni* including 6.7% (4/60) dried samples and 15.2% (20/132) fresh samples. Three of these isolates also had indistinguishable genotypes to those isolates recovered from human clinical cases, which provides evidence to support the link between wild bird faeces in playgrounds and human campylobacteriosis [19].

The presence of contaminated reptile faeces in the environment is possibly another emerging source of campylobacteriosis. A recent study in the United States reported nine cases of infection with *C. fetus* subsp. *testudinum* subsp. *nov*. This is a recently discovered subspecies of *Campylobacter* that appears to have originated in reptiles [8]. This is supported by a study by Wang *et al.* that identified *C. fetus* in 6.7% (12/179) of reptile fecal samples collected from domestic and wild reptiles in Taiwan [58]. 

## 4. Density and Fate in Solids

Given the mesophilic and micro-aerophilic nature of many *Campylobacter* spp., this bacterium cannot survive outside the host for extended periods of time [9,21,59] and as such, densities found in human and animal (e.g., bovine) biosolids can be low [60,61] (see Table 1). The rapid inactivation of *Campylobacter* spp. was demonstrated by Sinton *et al.*, who investigated the survival of laboratory-cultured *C. jejuni* in bovine faeces on pasture [62]. This work showed that in comparison to other indicator organisms (*Escherichia coli*, fecal streptococci, enterococci and *Salmonella enterica*), the inactivation of *C. jejuni* was rapid, even when the water content remained above 80%. T_90_ values were 16 days in winter, 2.7 days in spring, 1.2 days in summer and 4.7 days in autumn. It was concluded that temperature, rather than desiccation influenced survival and that post-excretion exposure to oxygen diffusing into the pat may have also increased the inactivation. Follow-up work by Gilipin *et al.* [23] also found that *Campylobacter* spp. naturally present in cow faeces exhibited a similar inactivation rate to that previously determined by Sinton *et al*. using laboratory-cultured strains [62]. Both studies are consistent with earlier work by Nicholson *et al.*, who monitored the inactivation of *Campylobacter* spp. during the land application of farm yard manure to different soil types. At 15–20 °C, this work showed that *Campylobacter* spp. survived for up to 8 days after application to sandy arable soils and 8–32 days for clay loam grassland soils [63].

**Table 1:** Density of *C. jejuni* in various sources of excrement.

Source	Density	Units	Reference
Poultry bird faeces	10^5^ to 10^8^	CFU g^−1^ of faeces	[31]
Domestic dogs	<10^3^–10^6^	Copies g^−1^ of faeces	[52]
Wastewater	1.9–3.2	Log_10_ 100 mL^−1^	[64]
	<10^4^	Copies mL^−1^	[61]
Sewage sludge	1 × 10^5^	L^−1^	[12]
	278	MPN g^−1^	[59]
	8.8 (±4.1) ×10^4^ – 3.9 (±0.9) ×10^5^	target gene copies/g of sludge wet weight	[61]
	1.5–4.4 (mean = 2.4)	Log_10_ 100 mL^−1^	[64]
	10^3^–10^5^	100 mL^−1^	[12]
Infected humans	10^6^–10^8^	g^−1^ of faeces	[65]
Cattle	<10^5^	Copies g^−1^ of faeces	[66]

More recently a study has demonstrated that *Campylobacter* spp. is able to persist and survive within compost, a relatively hostile environment, for up to 10 months. This study showed that the bacteria were able to survive within faeces from both untreated cattle and cattle treated with the antibiotics chlortetracycline and sulfamethazine. The antibiotics were added to cattle feed to improve weight gain, feed efficiency and to aid in the prevention of liver abscesses, bacterial diarrhoea, foot rot and bovine respiratory disease. Of the ingested chlortetracycline, 75% was excreted in cattle faeces; however, this had no effect on the presence and viability of *Campylobacter* spp. [35]. 

The discrepancy in survival noted between research by Sinton *et al.* [62] and Inglis *et al.* [35] could be attributed to the difference in culture and molecular identification techniques. Culture techniques often underestimate the number of bacteria in an environmental sample; the latter study used quantitative PRC in conjunction with ethidium monoazide treatment (EMA) which ensured only intact cells are amplified [67].

### 4.1. Storage of Biosolids and Manure

Ahmed and Sorensen [68] investigated the impact of temperature on inactivation rates of *C. jejuni* during the storage of dewatered biosolids. Compared to *S. Typhimurium*, *C. jejuni* was found to be more sensitive to heat. Results showed that 4.5 to >6 log_10_ reduction of *C. jejuni* occurred within one day at 49.5 °C. At cooler temperatures of 22 and 38 °C, 11 and six days were required to achieve a comparable log_10_ reduction. At 5 °C, *C. jejuni* were more persistent, whereby a 2 log_10_ reduction was observed within a 62 day period. Similar observations were noted during the storage of farmyard manure by Nicholson *et al*. This study reported a survival time of 2–5 days for both turned and unturned stockpiles of solid farmyard manure, when temperatures greater than 50 °C were obtained [63]. In contrast, *Campylobacter* spp. were shown to survive in stored slurries at temperatures of *ca* 15–20 °C for up to 32 days. In both instances, the inactivation of *Campylobacter* spp. was more rapid than *Salmonella*, *Listeria* and *E. coli*.

### 4.2. Anaerobic: Mesophilic Anaerobic Digestion

In contrast to aerobic environments, *C. jejuni* has been shown to be more resilient than indicator organisms during the anaerobic digestion of biosolids. During primary mesophilic anaerobic digestion of sewage sludge, Horan and his colleagues found no inactivation of *C. jejuni* within a operational period of 22 days and at a temperature of 35 °C [69]. Under the same conditions, a log removal of 1.66 was observed for *E. coli*, 2.23 for *Listeria monocytogenes* and 2.23 for *Salmonella senftenberg* respectively. The same study showed that a log_10_ *C. jejuni* removal of only 0.36 was achieved during a secondary sludge digestion stage, at cooler temperatures of 15 °C. This data is consistent with Kearney *et al.* who reported limited inactivation (1 log_10_ reduction in 793 days) of *Campylobacter* spp. during mesophilic anaerobic digestion [70].

## 5. Sewage

Studies have shown *Campylobacter* spp. to be ubiquitous in sewage [59,71,72,73,74]. Raw wastewater numbers of *Campylobacter* spp. can vary between 2–5 log_10_ per·L^−1^ [61,72].

The effectiveness of a sewage treatment plants in reducing *Campylobacter* spp. numbers during regular conditions depends on the complexity of the plant. Research by Arimi *et al.* found that primary sedimentation was able to reduce *Campylobacter* spp. numbers by 78%. Such a reduction is due to the fact that most micro-organisms are bound with solids [71]. In comparison to commonly used indicator organisms such as *E. coli*, Wéry *et al.* found *C. jejuni* were more resistant to biological treatment [61]. Nevertheless, biological treatment processes such as trickling filters, activated sludge plants and oxidation ponds are able to achieve a decimal reduction within the order of 0.6–2 log_10_ units [59,72,75].

Treated effluent is either pumped in to marine waters [21] or reused for irrigation [76,77] and the effective removal of *Campylobacter* spp. by tertiary treatments is crucial in preventing contamination of potential sources of human exposure [78]. 

## 6. Water Sources/Surface Waters

As previously mentioned, *Campylobacter* spp. are unable to grow outside warm blooded hosts [9] and can maintain long term contamination of environmental water sources [79]. There is great debate over the length of time that *Campylobacter* spp. can survive outside a host. Bushwell *et al.* demonstrated that *Campylobacter* spp. could survive only up to 29 days in water [80]; however, Rollins *et al.* demonstrated that they could survive for over 120 days in water [81]. The discrepancies between studies are thought to be caused by the bacteria entering a viable non-culturable state that prevents detection via traditional culture based techniques [81,82].

Environmental water sources have been associated with human campylobacteriosis. *Campylobacter* spp. have been isolated from a variety of environmental water sources including rivers [83], lakes [84], streams [85] and coastal waters [86]. Environmental waters can become contaminated through a variety of mechanisms including direct contamination with animal and avian faeces, agricultural run-off from farms, small holdings, slaughterhouses, slurry that is sprayed onto land and sewage effluent [21,83].

The incidence of campylobacteriosis follows a similar trend to most waterborne diseases, with a peak in incidence observed during late spring and early summer months [87]. This pattern, however, is not supported by quantitative studies of surface water which have shown there to be higher numbers of *Campylobacter* spp. present in surface waters during the winter months when compared to summer months. It has been postulated that the decrease in *Campylobacter* spp. numbers in the summer is due to elevated levels of UV light and higher temperatures [12]. 

In open waters, solar radiation is widely considered to be a dominant inactivation agent, which directly reduces the density of pathogens [88,89]. Sinton and his colleagues showed that sunlight insolation needed for 90% inactivation (S_90_ MJ·m^−2^) of *Campylobacter* spp. in river water and seawater augmented with STP effluent was 1.7 and 1.4 MJ m^−2^ respectively [62]. Under strong, optimal sunlight conditions in Spain and Bolivia (maximum global irradiance of ~1,050 W·m^−2^), Boyle *et al.* demonstrated that a 4 log_10_ unit reduction of *C. jejuni* was achieved in a short timeframe of 20 min, when using transparent water containers [90]. This resulted in a rapid solar inactivation rate (S_90_) of only 7 (±3) kJ/m^−2^. Under dark conditions, Sinton *et al.* found that T_90_ (time needed for 90% inactivation) values were a short 35 and 82.6 h for river water and seawater microcosms augmented with STP effluent. Inactivation of enteric microbes in dark, natural waters can largely attributed to the predatory, lytic, and grazing effects [91]. In the study by Sinton *et al.* the dark inactivation rates (T_90_) were thought to have been accelerated by the presence of residual predatory microbiota within the STP effluent. The significance of predatory biota on the survival of *Campylobacter* spp. in lake water was also noted in the work by Korhonen and Martikainen [92]. 

### 6.1. Groundwater

Groundwater is rarely considered as a reservoir for pathogenic microorganisms. The soils that bacteria must pass through to reach the surface generally function to attenuate microorganisms through simple filtration. Groundwater reaches the surface at boreholes, wells, springs and seeps. It is frequently used for irrigation and drinking water for livestock on farms [12,93]. However contaminated groundwater has been identified as a source of campylobacteriosis associated with outbreaks in poultry flocks [94] broiler chickens [95] and dairy farms [93]. Subsurface aquifers of groundwater provide favourable conditions for *Campylobacter* spp. survival, including constant temperature all year round, and protection from UV and desiccation. Similar conditions are found in larger groundwater aquifers that are used to deliver water to big cities and could be a potentially overlooked vehicle for transmission of campylobacteriosis in animals reared for food [12].

### 6.2. Drinking Water

Drinking water has been implicated in a number of sporadic outbreaks of campylobacteriosis [82,96,97,98,99]. Predominantly outbreaks are a result of consumption of untreated or contaminated water [12]. Private water supplies (PWS) are the number one source of contaminated drinking water. In England and Wales the majority of PWS are springs, boreholes and wells and a review conducted between 1992 and 2003 identified PWS as the source of 13 outbreaks of human campylobacteriosis [100]. Rainwater tanks specifically have been identified as a source of contamination in reported outbreaks of campylobacteriosis [101,102,103]. One study conducted in Australia found that 44% (12/27) rainwater tanks used for drinking water contained *Campylobacter* spp. with the major source of considered to be avian or possum faecal matter [99].

### 6.3. Campylobacter *spp.* and Water Disinfection

The contamination of water with *Campylobacter* spp. has been identified as both a direct source of contamination and an indirect source through the cross-contamination of drinking water and carcasses at processing plants. Studies have found that common disinfection processes designed to remove coliform bacteria from drinking water were sufficient to eliminate *Campylobacter* spp.*.* For example, Blaser *et al.* demonstrated that under a range of temperature and pH conditions, *C. jejuni* was more susceptible to chlorine and monochloramine disinfection than *E. coli.* This study showed that 2 log_10_ inactivation of *C. jejuni* was achieved after 15 min of contact with 1.0 mg L^−1^ of monochloramine or 5 min of contact with 0.1 mg L^−1^ of free chlorine [104]. Review data by Hijnen *et al.* also showed that *C. jejuni* was more susceptible to UV than *E. coli*. This review data showed that UV fluence of 3, 7 and 10 mJ/cm^2^ were required to permit a *C. jejuni* inactivation log_10 _ credit of 1, 2 and 3 respectively, and that *E. coli* required an additional dose of *ca.* 2–4 mJ/cm^2^ to reach an equivalent inactivation credit. These results suggest that disinfection procedures which are commonly based on meeting *E. coli* targets are adequate to eliminate *C. jejuni*. This is supported by the absence of campylobacteriosis outbreaks associated with properly disinfected water [104].

A recent study has demonstrated a decrease in the susceptibility of *Campylobacter* spp. to disinfection. Snelling *et al.* demonstrated the ability of *C. jejuni* to become internalised by the waterborne protozoa *Tetrahymena pyriformis* and *Acanthamoeba castellanii* within broiler drinking water systems. This study also demonstrated that internalised *Campylobacter* spp. were significantly more resistant to disinfection than their planktonic counterparts [105]. The formation of protozoan cysts have been shown to provide internalised bacteria protection from a range of other unfavourable environmental conditions, such as low nutrients, heat, desiccation and osmotic stress. Internalised bacteria within the protozoa cysts are also provided with a method for further contamination as protozoan cyst can be dispersed through the air [106].

## 7. Detection Methods

The difficulties in identifying environmental reservoirs of *Campylobacter* spp. are amplified by inaccuracies surrounding detection methods. The three main methods of identification are: traditional culture method using selective agar, membrane filtration onto blood agar and real time PCR [107,108,109]. The most common selective agar used is charcoal cefaperazone desoxycholate agar containing 32 mg/L cefoperazone. Plates are incubated at 37 °C for two days in anaerobic campy jars. Selective culture is a quick, cheap and effective method for identifying *C. jejuni* and *C. coli* from faecal samples [108]. However plates are often overgrown by faster growing microorganisms and this method does not identify the less common species. Filtering samples through a cellulose triacetate membrane with 0.45 mm pores onto blood agar separates the *Campylobacter* spp. from other larger bacteria which could overgrow the agar. This method is able to detect all cultivable *Campylobacter* spp. as there is no antibiotic used in the medium. Plates are also able to be incubated for longer without being overgrown which allows for the isolation of slower growing species [108]. The sensitivity of the membrane filtration technique is less than the selective culture and both of these techniques are less effective when applied to isolating *Campylobacter* spp. from water samples [96]. The problems associated with isolating *Campylobacter* spp. from water samples it the bacteria’s tendency to enter a viable but non-culturable state in unfavourable environmental conditions. This includes starvation but also physical stress which can occur during the process of sampling and storing cells [110]. 

Real time PCR allows for the identification of *Campylobacter* spp. to the species level and results can be achieved in one day. However it does not provide an isolate for further research, it is expensive and also highly labour intensive [108]. Detection and enumeration of viable but non-culturable cells is achieved using real time PCR. The problem with this method is that total counts can be overestimated due to the amplification of non-viable or killed cells. DNA within environmental samples can be very stable and is able to persist for extended lengths of time [67]. Novitsky [111] demonstrated that in marine sediment and salt water only 60%–70% of the DNA from killed organisms was in 14 days. This is an important issue in detection due to the varying lifespan of *Campylobacter* spp. that is dependent on both the strain and a range of environmental conditions such as temperature and free oxygen [12]. Techniques such as the use of ethidium monoazide (EMA) can be used in tandem with real time PCR to ensure only whole intact cells are amplified. When exposed to light EMA binds to any DNA that is not protected by a cell membrane and hence prevents amplification and enumeration. EMA has been optimised for use in water but is yet to be optimised in other environmental samples and is still not widely applied [67,111,112,113]. 

rRNA specific fluorescently tagged probes have also been used to examine *Campylobacter* spp. within aquatic biofilms. This method is valuable in examining *Campylobacter* spp. *in situ* within a biofilm. They are not effective for detection as they does not allow for enumeration and specificity can be significantly reduced by background fluorescence [80]. 

### 7.1. Molecular Typing

Molecular typing is an emerging tool which has been used to enhance many epidemiological studies. It enables the source of a patient’s *Campylobacter* spp. isolate to be identified based on its genome [3]. Multi-locus sequence typing (MLST) of clinical isolates and food and environmental isolates has been used as an epidemiological tool to estimate the relative importance of each source of human campylobacteriosis in New Zealand [17], Finland [114], Canada [115] and Scotland [116]. Whole genome sequencing (WGS) is another nucleotide typing tool, the benefit of WGS is that it has the highest discriminatory power and can differentiate between a single base pair. The expensive nature and difficulties associated with managing large databases of information, means that it’s use is still not widespread. It remains to be seen if WGS can be used for large scale epidemiological surveillance or if there will continue to be a need for inexpensive front line sub-typing methods [117]. 

### 7.2. Comparison to Faecal Indicators

Indicators of faecal contamination such as faecal coliforms and streptococci are often used as indicator organisms for the presence of faecal pathogens [12]. However Carter *et al.* isolated *Campylobacter* spp. from a range of natural water sources in central Washington including ponds, lakes and mountain streams. Microbiological plate counts were conducted and the results established that there was a lack of significant correlation between the occurrence of *Campylobacter* spp. densities and faecal coliforms, total coliforms, faecal Streptococci and heterotrophic bacteria. This lack of correlation suggests that further studies are needed to identify possible indicator organisms which could predict the presence of *Campylobacter* spp. in water [85].

This review suggests that in comparison to indicators, *Campylobacter* spp. can potentially survive longer in environments that are low in oxygen (e.g., in stockpiled slurries and anaerobic digesters) and vice versa in aerobic environments (e.g., land application of solids, activated sludge) (see Table 2).

**Table 2:** *Campylobacter* inactivation by environmental and treatment processes compared to faecal indicator organisms.

Barrier	Source	Conditions	Inactivation	Units	Comparative inactivation	Reference
Solar inactivation	River water + STP effluent	Natural sunlight conditions	1.65–1.68	S_90_ (MJ·m^−2^)	Higher than *E. coli* and *S. enterica*	[62]
	Sea water + STP effluent	Natural sunlight conditions	1.28–1.38			
	Transparent water bottles	Optimal sunlight conditions	7 (±3)	S_90_ (kJ·m^−2^)	Higher than *S. epidermidis, E. coli* and *Y. enterocolitica* and *B. subtilis*	[90]
UV treatment	Potable water	UV fluence of 3 mJ/cm^2^	1	Log_10_	Higher than *E. coli*	[118]
		UV fluence of 7 mJ/cm^2^	2			
		UV fluence of 10 mJ/cm^2^	3			
Free chlorine	Potable water	0.1 mg·L^−1^ after 5 min contact	2	Log_10_	Higher than *E. coli*	[104]
Monochloramine	Potable water	1.0 mg·L^−1^ after 15 min contact	2			
Primary sedimentation	Sewage		78	%		[71]
Trickling filters	Sewage		0.6			[64]
Activated sludge + settling	Sewage		1–2.5	Log_10_	Lower than *E. coli*	[61]
			1			[64]
Dark inactivation	Unfiltered lake water	14 days 4 °C	100	%	Higher than *E. coli*	[92]
		8 days at 25 °C	100	%		
	0.2 µm filter lake water	27 days at 4 °C	100	%		
		4 days at 25 °C	100	%		
	River water + STP effluent	120 L chambers	82.6	T_90_ (hours)	Higher than *E. coli* and *S. enterica*	[62]
	Seawater + STP effluent	120 L chambers	35	T_90_ (hours)		
Storage	Human biosolids	49.5 °C for 1 day	4.6–>6	log_10_	Higher than *S. Typhimurium*	[68]
		38 °C for 6 days	>6			
		22 °C for 11 days	>6			
		5 °C for 62 days	2			
	Farmyard manure	4 days at 50 °C	3	log_10_	Higher than *Salmonella*, *Listeria* and *E. coli*	[63]
		32 days 15–20 °C	3			
		4 and 17 °C for 112 days	0	log_10_	Lower than *E. coli*, *L. monocytogenes*, *Yersinia enterocolitica* and *S. typhimurium*	[70]
Land application	Farmyard manure	Sandy arable soils. 4–8 days at 11–20 °C	>3	log_10_	Higher than *Salmonella*, *Listeria* and *E. coli*	[63]
		Clay loam grassland soils. 8–32 days at 15–20 °C	2			
	Bovine manure applied to pasture	Winter	16	T_90 _(days)	Higher than *E. coli*, fecal streptococci, enterococci and *S. enterica*	[62]
		Spring	2.7			
		Summer	1.2			
		Autumn	4.7			
Anaerobic digestion	Human biosolids	22 days at 35 °C	0	log_10_	Lower than *E. coli*, *L. monocytogenes* and *S. senftenberg*	[69]
		25 days at 15 °C	0.36			
	Cattle slurry	793 days at 35 °C	1	log_10_	Lower than *E. coli*, *L. monocytogenes*, *Yersinia enterocolitica* and *S. typhimurium*	[70]

## 8. Conclusions

This paper provides a review of environmental sources of human campylobacteriosis and a preliminary tool for risk assessment. *Campylobacter* spp. are present in a range of environmental reservoirs, which include faeces of wild and domestic animals and municipal sewage. Many of these are a direct cause of human infection but also cross-contaminate other environments such as recreational and source waters, which have also resulted in human infection. Our review shows that the foodborne pathway of human *Campylobacter* spp. exposure has previously been overestimated and can be reduced significantly by providing consumers with greater information on the risk of raw meat and correct handling procedures. Importantly, further research and greater consideration of non-foodborne environmental reservoirs as a source of campylobacteriosis is required. This requires a better understanding of the fate and transport of *Campylobacter* spp. in the environment to better develop exposure assessment. However, to facilitate this, standard detection methods of *Campylobacter* spp. need to be established and tailored to the environmental source sampled. This is especially important due to the lack of correlation between common faecal indicators organisms and *Campylobacter* spp., meaning that they cannot be used to predict the presence or fate of *Campylobacter* spp. in the environment. Recently, our understanding of campylobacteriosis epidemiology has increased due to the development of molecular typing methods to identify sources of infection. The future application of these molecular typing methods, particularly WGS, for epidemiological studies will continue to improve our understanding of transmission routes and improve management strategies. 
